# Research on the Formability of 2A12 Aluminum Alloy Sheet During High-Speed Hot Gas Bulging

**DOI:** 10.3390/ma19102000

**Published:** 2026-05-12

**Authors:** Zichen Kang, Yingguang Zhao, Haochen Zhao, Yezhou Wang, Gaoning Tian, Cong Zhao, Jiangkai Liang, Xixing Qian, Yanli Lin, Zhubin He

**Affiliations:** 1 State Key Laboratory of High-Performance Precision Manufacturing, School of Mechanical Engineering, Dalian University of Technology, Dalian 116024, China; 18537716563@mail.dlut.edu.cn (Z.K.); 1754483197@mail.dlut.edu.cn (Y.Z.); jayfaily@mail.dlut.edu.cn (H.Z.); wangyezhoudut@163.com (Y.W.); sdtctgn@mail.dlut.edu.cn (G.T.); zhaocong9696@163.com (C.Z.); xixingqian@dlut.edu.cn (X.Q.); linyanli@dlut.edu.cn (Y.L.); 2 AVIC Xi’an Aircraft Industry (Group) Company Ltd., Xi’an 710089, China; 15535368969@163.com

**Keywords:** 2A12 aluminum alloy sheet, high-speed hot gas bulging, rapid inflation/deflation, formability, deformation behavior

## Abstract

In response to the growing demand for complex thin-walled lightweight alloy components in the automotive and aerospace industries, this study investigates the limitations of traditional gas pressure forming technologies. Using 2A12 aluminum alloy thin sheets as the research material, hot high-speed gas bulging experiments were conducted to study the effects of rapid inflation and rapid deflation processes on the forming accuracy, wall thickness, and strain distribution of bulged components. This aims to provide guidance for theoretical research and validate the superiority of the rapid deflation process. The results show that: (1) When forming cup-shaped components at 400 °C, the die-fitting degree of the component formed by the rapid deflation process reaches 89.5% and the minimum corner radius is 2.5 mm. Overall, the forming accuracy of this process is significantly superior to that of the rapid inflation process. (2) Within the temperature range of 400–450 °C, the rapid deflation process successfully formed a spherical-bottom component with a depth of 30 mm, overcoming the cracking defects induced by localized cooling and non-uniform temperature fields in the rapid inflation process, thereby improving the forming limit. (3) Under consistent conditions, the wall thickness uniformity of the sheet formed by the rapid deflation process is significantly higher than that of the sheet formed by rapid inflation, and the wall thickness uniformity improves with increasing temperature. Future work is expected to further enhance the repeatability and stability of forming accuracy and the forming limits of extreme geometries by further optimizing process parameters and expanding the material applicability range. This will provide practical technical support for the manufacturing of lightweight, high-performance aerospace equipment and automotive components.

## 1. Introduction

The demand for lightweight, high-performance, and complex thin-walled metal components in high-end equipment sectors such as aerospace and automotive industries has become increasingly urgent. Such components account for more than 80% of the structural parts in launch vehicles [1,2] and are predominantly manufactured from low-density, high-specific-strength materials, including aluminum, magnesium, and titanium alloys [3,4]. These components are typically characterized by large overall dimensions, numerous local geometric features, and thin wall thicknesses. However, these lightweight alloys exhibit poor plasticity at room temperature, making it difficult to directly form complex geometries [5,6,7]. Moreover, the forming process must simultaneously satisfy stringent requirements for high-dimensional accuracy, geometric complexity, and mechanical performance. Conventional forming techniques are prone to defects such as wrinkling, cracking, and non-uniform wall thickness, which severely limit the efficient and precise manufacturing of such components and have become a critical bottleneck in industrial development [8,9,10].

To address the forming challenges of complex components made from lightweight materials, high-temperature forming technologies are widely employed in industry. By elevating the forming temperature, the deformation resistance of the material is reduced and its plasticity is significantly improved [11,12,13,14,15]. From a metallurgical perspective, the elevated temperatures utilized in these mechanical forming processes activate specific microstructural phenomena. Thermally activated mechanisms, such as enhanced dislocation slip, dynamic recovery (DRV), and dynamic recrystallization (DRX), become dominant [16,17,18]. These phenomena critically reduce the material’s flow stress and inhibit the formation of micro-voids, thereby enabling complex geometric transformations that are fundamentally impossible under room-temperature conditions. This microstructural evolution and dynamic softening behavior are highly consistent with the findings reported in [19,20] under elevated temperature conditions. According to differences in deformation rate, existing high-temperature forming methods can be broadly classified into two categories [21]: (1) Superplastic forming (low-speed forming): This process operates at elevated temperatures, where the blank undergoes gradual deformation under a strain rate lower than 10^−2^ s^−1^ and a gas pressure below 1.0 MPa [22]. The main advantages of this method include stable forming quality and strong adaptability. However, the forming cycle for a single component typically requires several hours or even longer, resulting in extremely low production efficiency. In addition, both the die and the formed part must be cooled before demolding [23], making it unsuitable for large-scale production. (2) High-pressure gas bulging (rapid forming): In this process, rapid heating is achieved through various heating methods, and the blank is deformed within a short time under high gas pressures of 10 MPa or higher and relatively high strain rates [24]. This method offers substantially higher production efficiency than superplastic forming and is therefore suitable for the mass production of lightweight alloy components [25]. Nevertheless, when high-pressure gas at room temperature is introduced into a high-temperature die cavity, localized rapid cooling of the blank is likely to occur, resulting in a non-uniform temperature field. Furthermore, gas is typically supplied through localized inlet ports, leading to a non-uniform pressure distribution. For components with complex geometries, it is difficult to optimally arrange the gas inlet locations; the combined non-uniformity of temperature and pressure directly degrades forming quality and prevents the component from meeting precision requirements.

To overcome the limitations of existing high-temperature forming technologies, including non-uniform temperature and pressure distributions as well as insufficient forming accuracy for complex components, this study proposes a hot-state rapid gas depressurization forming technique. The underlying principle is to exploit the transient pressure differential to drive rapid deformation of the sheet under quasi-isothermal and quasi-isobaric conditions, thereby improving the uniformity of temperature and pressure distributions. This method offers significant advantages in enhancing corner filling capability, increasing the forming limit, and improving wall thickness uniformity, while simultaneously achieving high forming accuracy and production efficiency. At present, existing studies mainly concentrate on the optimization of individual processes, such as conventional high-pressure gas bulging or superplastic forming. However, systematic comparisons of deformation behavior between hot-state rapid gas depressurization forming and traditional high-pressure gas bulging are still lacking. In particular, there remains a significant research gap concerning the rapid deflation forming characteristics of 2A12 according to GB/T 3190-2020 [26] (equivalent to UNS/ASTM AA 2024 and to EN AW-AlCu4Mg1) aluminum alloy sheets within the temperature range of 350–450 °C and the underlying influence mechanisms of key process parameters, and the associated technologies have yet to be thoroughly explored and applied.

This study compares the hot-state high-speed gas pressure bulging process (rapid deflation) with the traditional gas pressure bulging process (rapid inflation). The specific objectives are systematically mapped as follows: (1) to evaluate the forming accuracy and corner-filling capability; (2) to investigate the effect of temperature on wall thickness distribution and uniformity; (3) to explore the forming limit capabilities under extreme depth conditions; and (4) to reveal the underlying strain distribution patterns of typical cross-sections. Future work may focus on key challenges such as complex cavity filling and forming of components with extreme dimensions, aiming to further optimize process adaptability and extend the technique to a broader range of lightweight alloys and component types. This will provide technical support for the efficient mass production of high-precision complex thin-walled components in the aerospace and automotive sectors, thereby promoting lightweight design and manufacturing upgrading of high-end equipment.

## 2. Principle of Hot Gas Bulging with Rapid Inflation/Deflation

**Figure 1:**
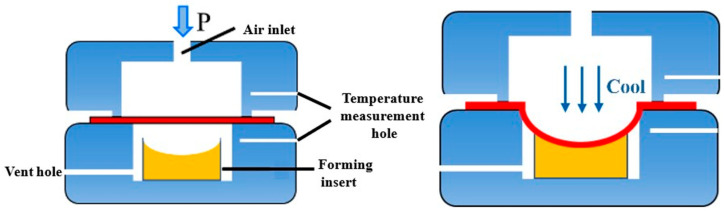
Schematic diagram of traditional gas forming.

The principle of the rapid plastic forming process (high-pressure gas bulging) is illustrated in Figure 1. After the upper and lower dies are closed, the sheet blank is held within the die for a certain period to reach the prescribed forming temperature. Subsequently, high-pressure gas at room temperature is introduced through the gas inlet, forcing the sheet to conform to the die cavity and form the desired geometry [27]. However, rapid gas pressure forming is limited by the non-uniform temperature and pressure fields caused by room-temperature gas, which deteriorates the sheet blank’s formability [28]. Consequently, for complex sheet metal components—particularly thin-walled structures—this process fails to achieve precise and rapid forming.

**Figure 2:**
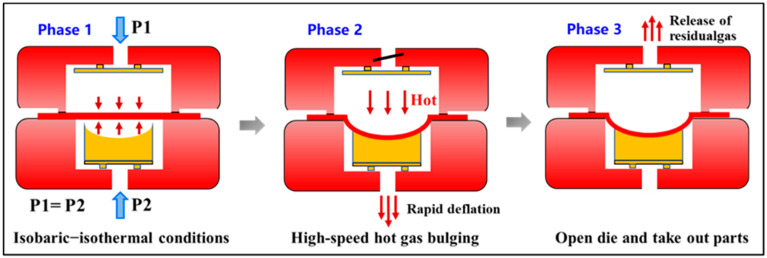
Principle schematic of the hot gas bulging with rapid deflation process.

The principle of the hot-state rapid gas pressure forming process is illustrated in Figure 2 and can be divided into three main stages. (1) High-pressure gas with identical pressure is simultaneously introduced into the upper and lower sealed chambers formed between the metal sheet and the sealing die, as well as between the metal sheet and the forming die, while the sheet is heated to the preset forming temperature. (2) The high-pressure gas in the sealed chamber between the metal sheet and the forming die is rapidly released, causing the sheet to undergo rapid bulging under the action of the high-pressure gas on the opposite side and to conform to the cavity of the forming die. (3) The gas within the chamber formed between the metal sheet and the sealing die is then discharged, after which the sealing die is opened to obtain the formed component.

In the hot-state rapid gas pressure forming process, the pressurization stage is independently controllable, thereby avoiding the difficulty of deformation control encountered in conventional direct high-pressure gas bulging, where pressure increase and sheet deformation occur simultaneously and evolve in a highly coupled and complex manner. Moreover, the temperature of the sheet remains essentially unaffected during forming. The blank is already at an appropriate forming temperature prior to bulging, and no external gas directly impinges on the sheet during deformation, preventing temperature fluctuations during the forming process. This effectively eliminates the adverse influence of unreasonable temperature variations induced by direct high-pressure gas injection in conventional processes, which can deteriorate bulging behavior. In addition, during bulging, the gas trapped between the sheet and the forming die is rapidly discharged within a very short time, leading to the rapid establishment of a pressure differential across the sheet. When this pressure differential reaches a sufficiently high level, the metal sheet undergoes rapid bulging deformation. Owing to the high deformation speed and elevated strain rate, metal sheets generally exhibit enhanced formability under these conditions, providing a favorable basis for forming complex components, particularly those involving large localized strains. However, this rapid deflation process inevitably presents certain limitations. The requirement for instantaneous gas release necessitates highly responsive and extremely durable pneumatic control systems, such as high-speed solenoid valves. Additionally, maintaining stringent gas sealing for the dual-chamber setup at elevated temperatures poses significant equipment and tooling challenges. Moreover, due to the extremely rapid and transient nature of the deformation, it is difficult to actively intervene or precisely adjust the strain path during the forming stage.

Furthermore, unlike traditional mechanical stamping or forging processes, hot gas bulging is fundamentally a flexible forming technique without a solid punch. Consequently, the effect of friction-generated heat at the die–sheet interface is negligible, preserving the nearly ideal quasi-isothermal conditions essential for uniform deformation.

## 3. Experimental Procedure

### 3.1. Material

The material used in this study was a rolled 2A12 according to GB/T 3190-2008 (equivalent to UNS/ASTM AA 2024 and to EN AW-AlCu4Mg1) aluminum alloy sheet with a thickness of 1.5 mm. Although 2XXX series alloys are typically utilized in the T6 condition for structural applications [29], the annealed (O-temper) condition was deliberately selected for the initial blank in this study. This maximizes the initial ductility required to accommodate the extreme plastic strains during high-speed bulging, with the understanding that post-forming heat treatments can be subsequently applied to recover high mechanical strength [30,31,32]. The material was in the annealed condition, and its chemical composition, determined using an optical emission spectrometer (X-ray fluorescence spectrometer S8 TIGER, SHINING 3D Tech Co., Ltd., Hangzhou, China), is listed in Table 1. To enable strain analysis of the bulged components, the sheet blanks were pretreated by printing a grid pattern on their surfaces prior to forming, facilitating subsequent deformation measurements. The bulging specimens had dimensions of 150 × 150 mm^2^, and their surfaces were required to be free from noticeable scratches or defects. A uniform grid of circular dots was produced on the sheet surface using an electrochemical etching technique. Each dot had a diameter of 1 mm with a spacing of 1.5 mm, ensuring clear and well-defined grid marks for accurate strain analysis.

### 3.2. Hot Gas Bulging Test

To systematically investigate the high-speed forming behavior of 2A12 aluminum alloy sheets under various conditions, experimental studies were conducted to compare the hot-state high-speed gas bulging process with the conventional gas bulging process. In addition, the deformation behavior of the hot-state high-speed gas bulging process under different temperatures and bulging pressures was examined. To systematically investigate the transient deformation behavior at different stages of the bulging process (early, intermediate, and final filling stages), varying bulging pressures of 6, 9, and 12 MPa were specifically selected for the flat insert experiments. The detailed experimental scheme is summarized in Table 2.

**Figure 3:**
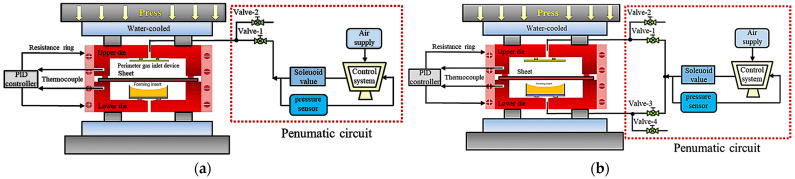
Circuit diagram of sheet hot gas bulging: (**a**) proposed hot gas bulging with rapid inflation; (**b**) proposed hot gas bulging with rapid deflation.

The experimental setup and gas-line configuration for the conventional rapid gas bulging process with rapid inflation are shown in Figure 3a. First, the die temperature was regulated using a PID controller. After heating to the target temperature, the die was held for 1 h to ensure thermal stabilization of the die cavity. The upper die was then opened, and the pretreated aluminum alloy sheet was quickly placed into the die. The press descended to close the die, and the blank-holding force was increased to approximately 15 MPa, followed by a holding period of 20 min. Valve 2 was closed and valve 1 was opened to rapidly introduce high-pressure gas. Two gas inlet configurations—central inlet and perimeter inlet—were employed. The perimeter gas inlet device in Figure 3 enables high-pressure gas to enter the die cavity along the die periphery, thereby prolonging the gas flow path within the cavity and mitigating the influence of room-temperature gas on the temperature field of the heated sheet. Moreover, the structure is detachable and can be reconfigured as needed to switch between perimeter rapid inflation and center rapid inflation. The pressurization time was set to 1 s, during which the sheet underwent rapid bulging and conformed to the forming insert. After bulging, the pressure was maintained for 3 s, after which valve 2 was opened to release the gas from the upper die cavity. Once the high-pressure gas was completely discharged, the press ascended to open the die, and the bulged component was promptly removed.

The experimental setup and gas-line configuration for the hot-state high-speed gas bulging process with rapid deflation are shown in Figure 3b. The initial steps of rapid deflation, including die temperature regulation, thermal stabilization, sheet placement, and the application of blank-holding force, are identical to those outlined in rapid inflation. The process then diverges from rapid inflation. Following the principle outlined in Section 2, the specific pneumatic operations were executed as follows: high-pressure gas was simultaneously injected into both cavities (valves 1 and 3 open, 2 and 4 closed) and held for 20 min. Subsequently, the lower cavity was rapidly depressurized within 1 s by closing valve 3 and opening valve 4, triggering the high-speed bulging. After a 3 s pressure-holding period, the upper cavity was exhausted (valve 2 open), and the formed component was demolded.

The bulged components were primarily subjected to contour analysis, strain analysis, and wall thickness evaluation. The surface of the formed components was scanned using an EinScan Pro 2X 3D optical scanner (Bruker AXS GmbH, Karlsruhe, Germany) to characterize the bulging contours. The die-fitting condition under different forming parameters was evaluated by calculating the spatial deviation between the component surface and the inner surface of the die. A smaller deviation value indicates a better die-fitting performance of the bulged component.

Strain analysis of the bulged components was performed using the ARGUS strain measurement system developed by GOM GmbH (Braunschweig, Germany). A grid pattern with identical size and geometry was printed on the surface of the original sheet using an electrochemical etching technique. After bulging, the formed components were photographed using a calibrated camera, enabling the acquisition of full-field strain distribution maps and the corresponding quantitative strain data over the entire deformation zone.

For wall thickness analysis, the bulged component was sectioned into two halves along the central axis, and thickness measurements were conducted along the cutting direction. A pointed micrometer with a measurement accuracy of 0.001 mm was used for thickness measurement. During measurement, the axis of the micrometer was carefully aligned with the axial direction of the bulged component. Each measurement point was measured three times, and the average value was taken as the final thickness result.

## 4. Results and Discussion

### 4.1. Forming Accuracy Analysis

**Figure 4:**
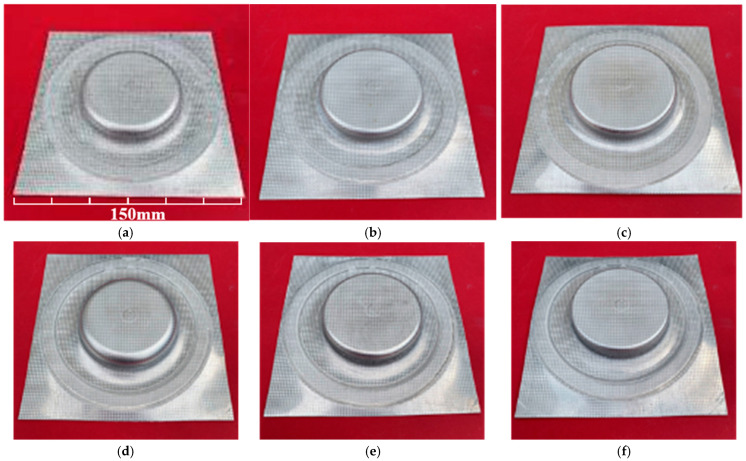
Bulged specimen by different bulging methods at deformation temperature of 400 °C and pressurizing rate of 6 MPa/s: (**a**) 6 MPa-perimeter rapid inflation; (**b**) 9 MPa-perimeter rapid inflation; (**c**) 12 MPa-perimeter rapid inflation; (**d**) 6 MPa-rapid deflation; (**e**) 9 MPa-rapid deflation; (**f**) 12 MPa-rapid deflation.

It should be noted (stressed out) that in this section regarding forming accuracy, the ‘center rapid inflation’ method is excluded from the comparative analysis. Preliminary observations revealed that central high-pressure gas injection causes severe localized cooling and excessive thinning at the apex, leading to premature fracture before the sheet can fully conform to the die corners. Therefore, a meaningful quantitative comparison of the ultimate die-fitting degree is only viable between the ‘perimeter rapid inflation’ and ‘rapid deflation’ processes. Figure 4 presents the bulged components formed by perimeter rapid inflation and rapid deflation at different bulging stages at 400 °C. A flat forming insert was used, with a pressurization rate of 6 MPa s^−1^, and bulging pressures of 6, 9, and 12 MPa were applied, representing different stages of the sheet bulging process. As shown in the figure, the die-fitting degree of the sheet gradually increases as the bulging process proceeds.

**Figure 5:**
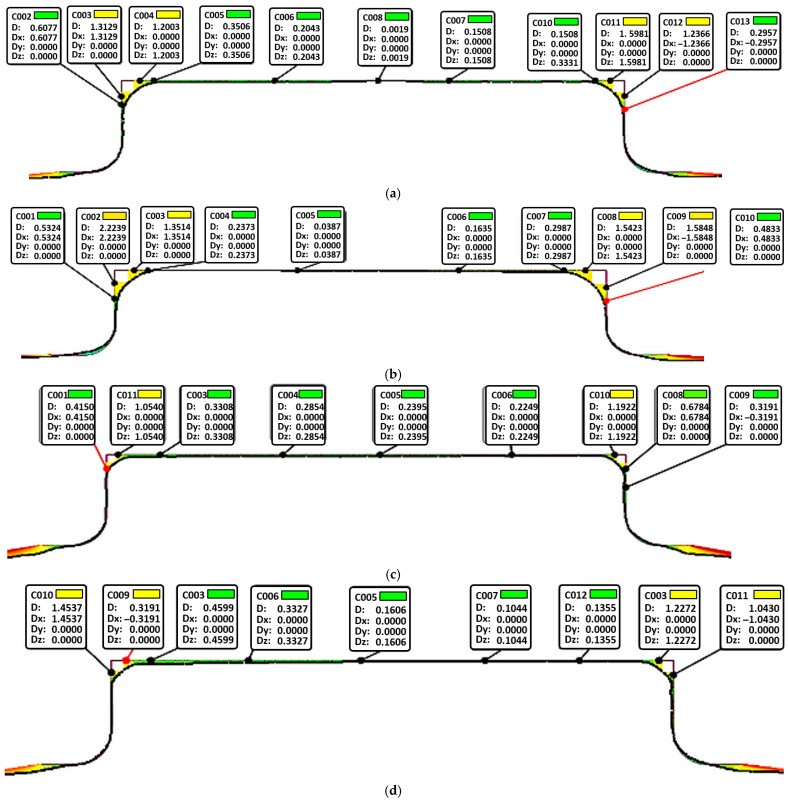

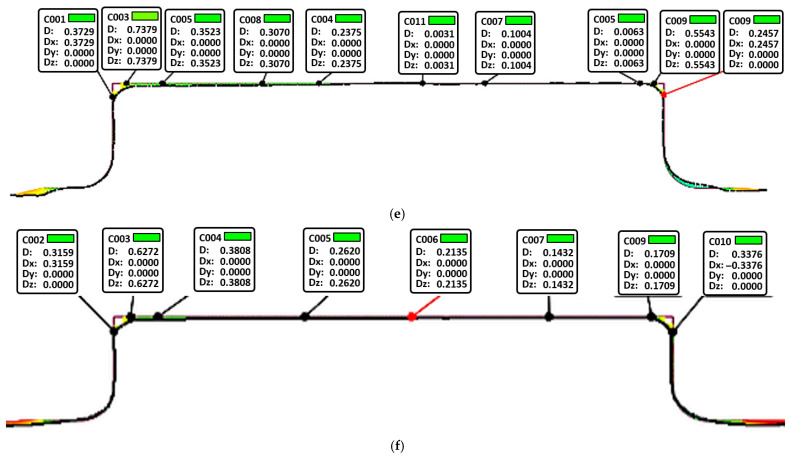
The profile by different bulging methods at deformation temperature of 400 °C and pressurizing rate of 6 MPa/s (The data in the figure represents the spatial deviation of the rounded area and the planar area. The smaller the deviation, the lower the film adhesion degree.): (**a**) 6 MPa-perimeter rapid inflation; (**b**) 6 MPa-rapid deflation; (**c**) 9 MPa-perimeter rapid inflation; (**d**) 9 MPa-rapid deflation; (**e**) 12 MPa-perimeter rapid inflation; (**f**) 12 MPa-rapid deflation.

The surfaces of the formed components were scanned using a 3D optical scanner, and the processed results are shown in Figure 5. The figure presents cross-sectional contour profiles of the die (upper) and the bulged component (lower). The data at each point in Figure 5 represent the spatial deviation between the surface of the bulged component and the inner surface of the die; a smaller deviation indicates better conformity of the formed part to the die. Under identical processing conditions, the die-fitting degree of the sheet increases progressively as the bulging process proceeds. The quantitative die-fitting degrees under different forming conditions are summarized in Table 3. Except for regions with extremely small fillet radii, the contour of the bulged component is in close conformity with the die profile. At the same bulging pressure, corresponding to the same bulging stage, the components formed by the rapid deflation process exhibit a higher die-fitting degree than those formed by the perimeter rapid inflation process, demonstrating the superiority of the rapid deflation technique.

**Figure 6:**
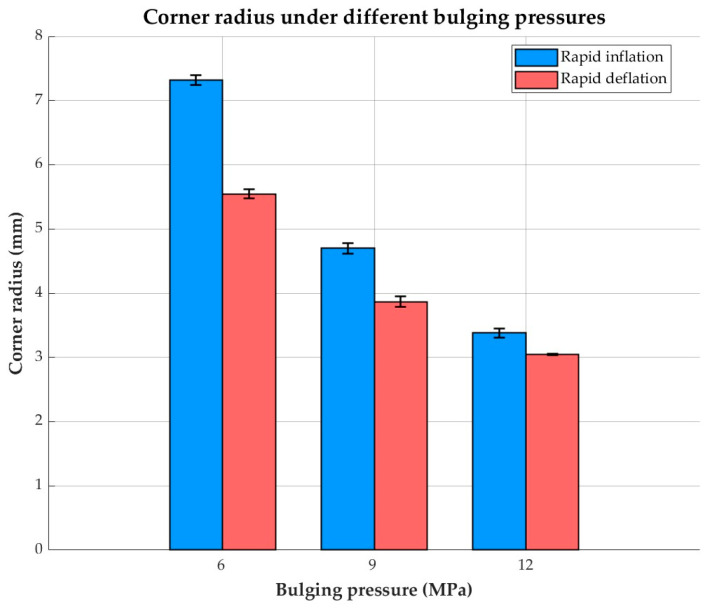
Corner radius by different bulging methods at deformation temperature of 400 °C.

The fillet radii of bulged components formed by perimeter rapid inflation and rapid deflation were measured and analyzed, as shown in Figure 6. For sheets formed using both rapid inflation and rapid deflation processes, the corner radius decreases as the pressure increases. The data in the figure are the average values obtained from three experiments, and the error bars represent the standard deviation. Under identical forming conditions, the components produced by the rapid deflation process exhibit smaller fillet radii. This indicates that the rapid deflation technique enhances the corner-filling capability of the sheet, resulting in higher forming accuracy. It demonstrates potential for forming fine features and provides a feasible approach for manufacturing complex thin-walled components.

### 4.2. Thickness Distribution Analysis

The wall thickness distribution of the formed specimens is closely related to the bulging method. Different bulging methods result in distinct wall thickness distribution characteristics. To systematically investigate the influence of different processes on wall thickness distribution, both rapid inflation bulging experiments and rapid deflation bulging experiments were conducted. In the rapid inflation bulging experiments, two gas inlet configurations were adopted: a central gas inlet and a perimeter gas inlet.

**Figure 7:**
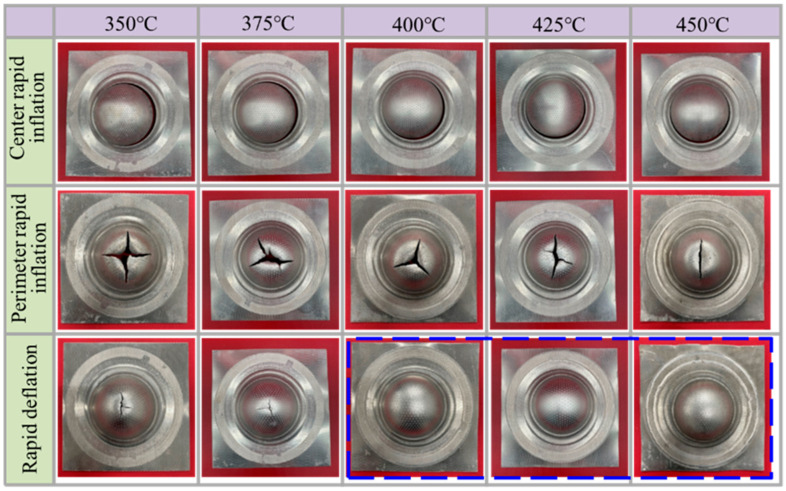
Bulged specimen with a bulging height of 30 mm at different temperatures.

Figure 7 shows the forming results and cracking defects of bulged specimens produced using different gas loading/unloading methods (central gas inlet, perimeter gas inlet, and rapid deflation) at a fixed bulging height of 30 mm and forming temperatures of 350, 375, 400, 425, and 450 °C. The results indicate that, under the central gas inlet condition, cracking occurs in the perimeter region of the specimens at all investigated temperatures. This behavior is mainly attributed to the inflow of room-temperature gas, which reduces the temperature of the central region and increases its deformation resistance, thereby shifting deformation toward the perimeter region and causing excessive thinning and cracking at the edges. Under the perimeter gas inlet condition, cracking is consistently observed in the central region of the bulged specimens. This is because the room-temperature gas lowers the temperature and plasticity of the perimeter region, leading to deformation concentration in the central area. Moreover, as the forming temperature increases from 350 to 450 °C, the cracking region gradually decreases. At 450 °C, cracks propagate along the rolling direction, indicating the enhancement of sheet plasticity with increasing temperature. In contrast, the rapid deflation process effectively reduces the influence of room-temperature gas on the temperature field of the sheet, resulting in a more uniform temperature distribution. At 350 °C, cracking still occurs in the central region due to insufficient overall plasticity; however, the cracking area is significantly smaller than that observed under conventional gas inlet methods. At 375 °C, the severity of cracking is further reduced. When the temperature increases to 400, 425, and 450 °C, the sheet can be successfully bulged to the target height without any cracking defects. This demonstrates the superior forming performance and process adaptability of the rapid deflation method.

**Figure 8:**
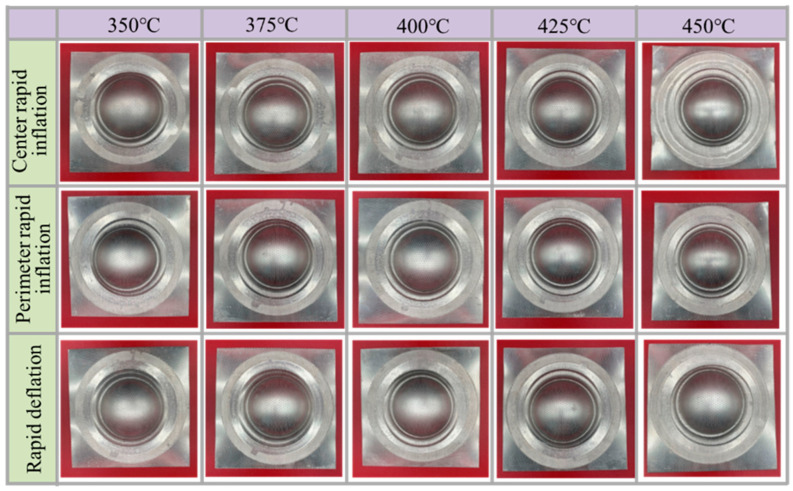
Bulged specimen with a bulging height of 20 mm at different temperatures.

Figure 8 shows the bulged specimens formed at temperatures ranging from 350 to 450 °C with a bulging height of 20 mm. Using both the rapid inflation process and the rapid deflation process, the specimens can successfully reach the target bulging height. For specimens with identical bulging profiles, the bulged parts were sectioned along the central region to analyze the wall thickness distribution in the central area.

**Figure 9:**
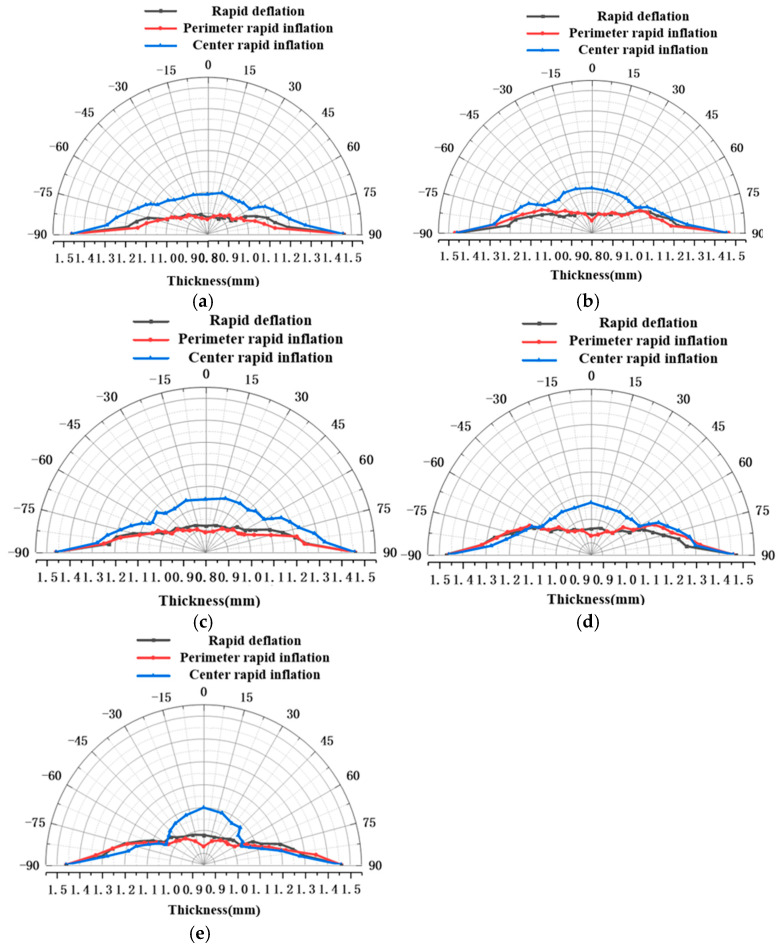
Wall thickness distribution at 20 mm bulging height under different temperature conditions: (**a**) 350 °C; (**b**) 375 °C; (**c**) 400 °C; (**d**) 425 °C; and (**e**) 450 °C.

Figure 9 shows the wall thickness distributions of bulged specimens formed to a height of 20 mm under the same heating temperature using different bulging methods. Different bulging methods affect the temperature distribution during forming, which in turn influences the deformation resistance across different regions of the sheet, leading to variations in forming performance and wall thickness distribution. Comparison of the wall thicknesses obtained using rapid deflation, central rapid inflation, and perimeter rapid inflation reveals that the two conventional inflation methods produce specimens with non-uniform wall thickness, whereas the rapid deflation process significantly improves thickness uniformity. Specifically, compared with the rapid deflation process, perimeter rapid inflation results in more pronounced thinning at the apex, and the thickness difference between the two methods increases with rising forming temperature. This is because the perimeter gas inlet lowers the temperature and increases the deformation resistance at the sheet edges, leading to greater deformation in the central region and consequently excessive thinning at the apex. In contrast, the central rapid inflation process exposes the sheet center directly to room-temperature gas, reducing the central temperature and increasing its deformation resistance. As a result, the deformation at the center is less than at the periphery, producing a non-uniform distribution characterized by a thicker apex and thinner edges. The rapid deflation process, by establishing a near-isothermal and near-isobaric forming environment and utilizing the pressure difference generated in the upper and lower die cavities during rapid gas release, avoids interference from room-temperature gas, ensures uniform temperature distribution across the sheet, promotes balanced overall deformation, and ultimately results in a more uniform wall thickness distribution in the bulged specimen. These findings regarding improved thickness uniformity align with trends observed in advanced superplastic forming studies (e.g., [34]), where isothermal conditions suppress localized necking. However, compared to conventional superplasticity, which relies on slow strain rates, the rapid deflation process proposed herein achieves comparable or superior uniformity within mere seconds, validating its efficiency advantage.

The formability of the sheet is closely dependent on the temperature conditions. Bulged specimens with identical bulging heights were produced at different temperatures using the hot-state rapid gas pressure forming process. These specimens were used to investigate the influence of temperature on the wall thickness distribution.

**Figure 10:**
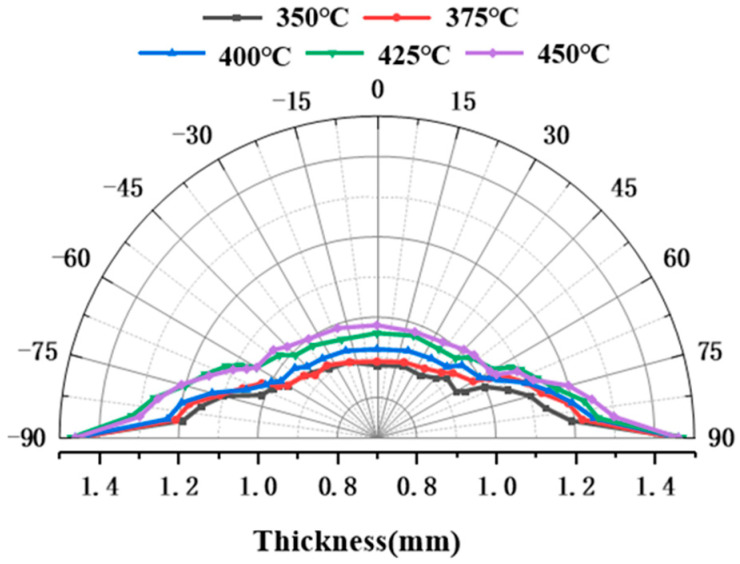
Wall thickness distribution at 20 mm bulging height under different temperature conditions.

**Figure 11:**
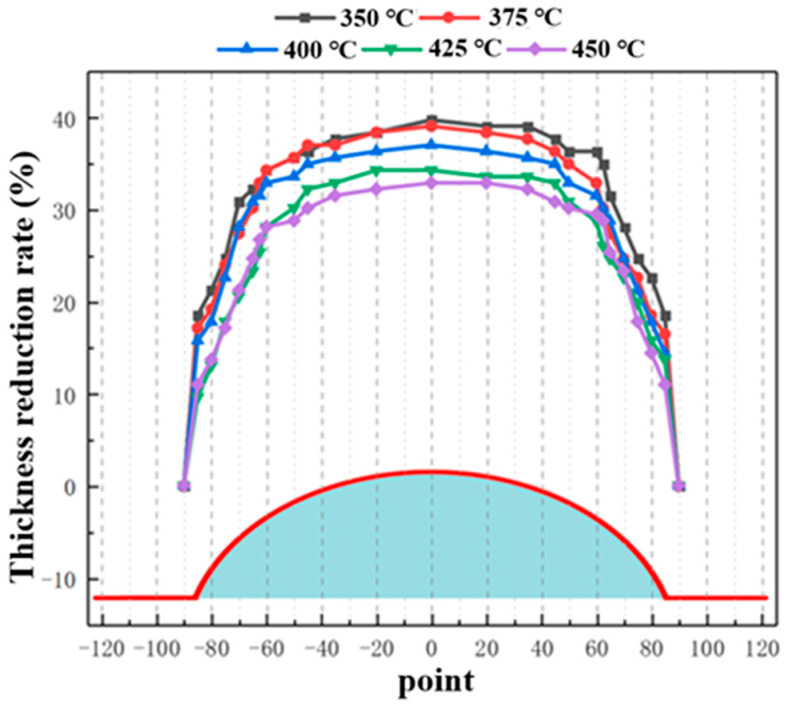
Thinning rate of wall thickness at 20 mm bulging height under different temperature conditions.

Figure 10 and Figure 11 present the wall thickness distributions and thickness reduction rates of bulged specimens formed under different temperatures using the hot-state rapid gas pressure forming process with rapid deflation. The key trends are as follows: as the forming temperature increases, the overall wall thickness of the specimens increases. The regions of maximum thinning are consistently located at the apex, which directly corresponds to the areas of largest deformation and strain. With increasing temperature, the maximum thickness reduction rate decreases. Furthermore, from a metallurgical perspective, elevating the forming temperature to the range of 400–450 °C triggers dynamic recovery (DRV) and partial dynamic recrystallization (DRX) within the 2A12 aluminum alloy. These thermally activated microstructural evolutions effectively annihilate the dislocations generated during rapid deformation, thereby mitigating the severe work hardening induced by the high-strain-rate forming process. This dynamic softening mechanism significantly reduces the local flow stress and postpones the onset of localized necking, ultimately optimizing the overall deformation uniformity and thickness distribution. For example, at 350 °C, the maximum variation in thickness reduction within ±60° of the apex is 5.51%, which decreases to 4.79% at 450 °C. This further confirms that increasing the forming temperature effectively enhances the uniformity of deformation in the bulged region.

**Figure 12:**
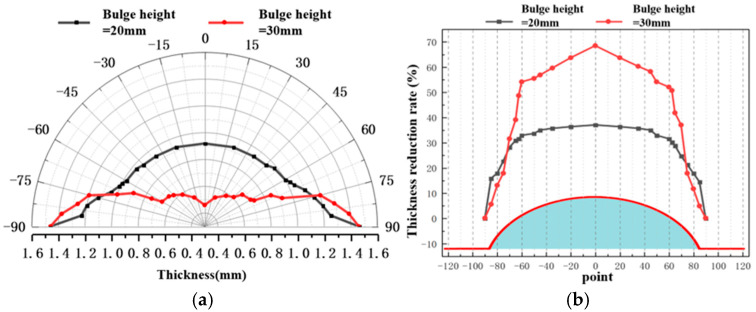
Wall thickness analysis of different bulging heights at 400 °C: (**a**) wall thickness distribution; (**b**) wall thickness reduction rate.

**Figure 13:**
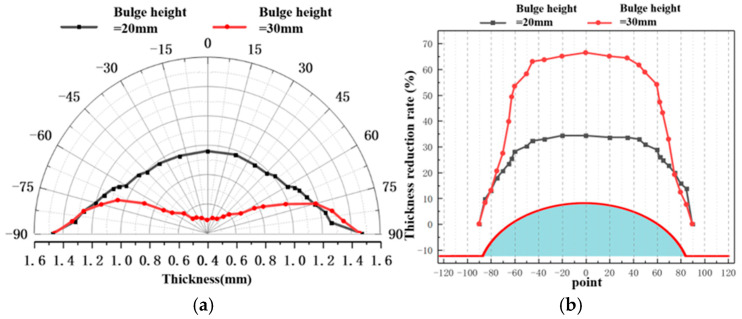
Wall thickness analysis of different bulging heights at 425 °C: (**a**) wall thickness distribution; (**b**) wall thickness reduction rate.

**Figure 14:**
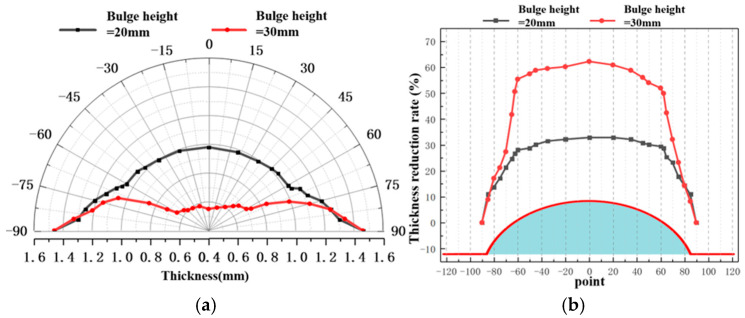
Wall thickness analysis of different bulging heights at 450 °C: (**a**) wall thickness distribution; (**b**) wall thickness reduction rate.

Figure 12, Figure 13 and Figure 14 present the wall thickness distributions and thickness reduction rates of sheets bulged at different temperatures and bulging heights using the hot-state rapid gas pressure forming process with rapid deflation. Overall, under the rapid deflation process, the wall thickness distribution, thinning rate, and uniformity of bulged sheets are primarily influenced by bulging depth and forming temperature. As the bulging depth increases from 20 mm to 30 mm, the wall thickness reduction becomes more pronounced under all temperature conditions, the thickness variation range widens, uniformity deteriorates, and the apex region (0° direction) consistently exhibits the maximum thinning. Conversely, as the forming temperature rises from 400 to 450 °C, the overall wall thickness increases, the thickness variation gradually decreases, and uniformity is significantly improved. This improvement is attributed, on one hand, to the high strain rate induced by the instantaneous pressure difference during the rapid deflation process, which enhances hardening at the sheet center and reduces thinning, and on the other hand, to the elevated temperature further intensifying strain rate hardening, thereby optimizing overall deformation uniformity [35].

### 4.3. Strain Distribution Analysis

**Figure 15:**
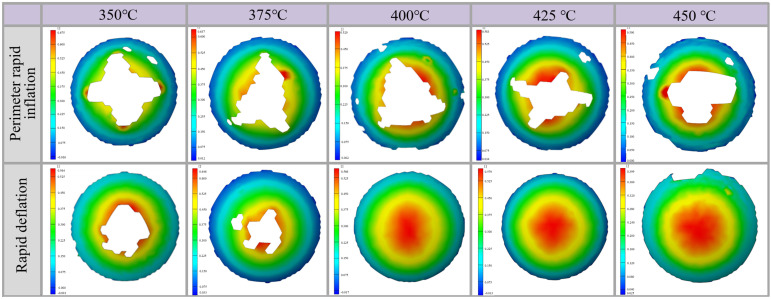
Strain distribution of bulged specimens with a forming depth of 30 mm under different bulging methods and deformation temperatures.

As established in the preceding thickness distribution analysis, the direct impingement of room-temperature gas at the center during ‘center rapid inflation’ drastically degrades local formability. This renders the method incapable of approaching extreme target depths (e.g., 30 mm) without catastrophic failure. Consequently, the strain distribution and cracking behavior analysis presented in this section centers on the comparison between the more capable perimeter inflation and the proposed rapid deflation processes. To investigate the effects of forming temperature and bulging method on the forming quality, strain distribution, and cracking behavior of hot-state high-speed gas-bulged components, a forming insert with a depth of 30 mm was used as the die, with pressurization/depressurization time fixed at 1 s and holding time at 3 s. Both perimeter rapid inflation and rapid deflation bulging processes were applied. Bulging experiments were performed at five temperature levels: 350, 375, 400, 425, and 450 °C. The experimental results are presented in Figure 15. It is also worth noting that due to the 20 min holding period prior to deflation (which effectively stress-relieves the initial blank), the observed deformations and strain states are entirely governed by the mechanical forming process driven by the transient pressure differential, rather than any pre-existing residual stresses. It should be noted that to maximally highlight the local strain gradient characteristics and the precise location of strain concentration under each specific condition, the strain scales in the full-field maps are independently automatically adjusted to their local maximums rather than using a unified global scale. Additionally, the ‘white zones’ absent on color mapping observed in some strain distribution images represent areas where the ARGUS system could not recognize the grid. This phenomenon is primarily caused by severe localized thinning culminating in macroscopic cracking or the severe detachment of the grid paint at the fracture boundaries.

As summarized in Table 4, when the temperature reaches 400 °C or above, the rapid deflation process can reliably achieve an extreme bulging depth of 30 mm without cracking, demonstrating enhanced adaptability to extreme deformation. By creating an approximately isothermal and isobaric environment, this process ensures uniform temperature and pressure distribution across the sheet, combined with strain-rate hardening effects [36]. It not only improves forming quality under extreme bulging conditions but also enables efficient forming under high-speed gas pressure loading. This extends the operational limits of the hot-state high-speed gas bulging process and provides a reliable technical foundation for the mass production of complex thin-walled components.

**Figure 16:**
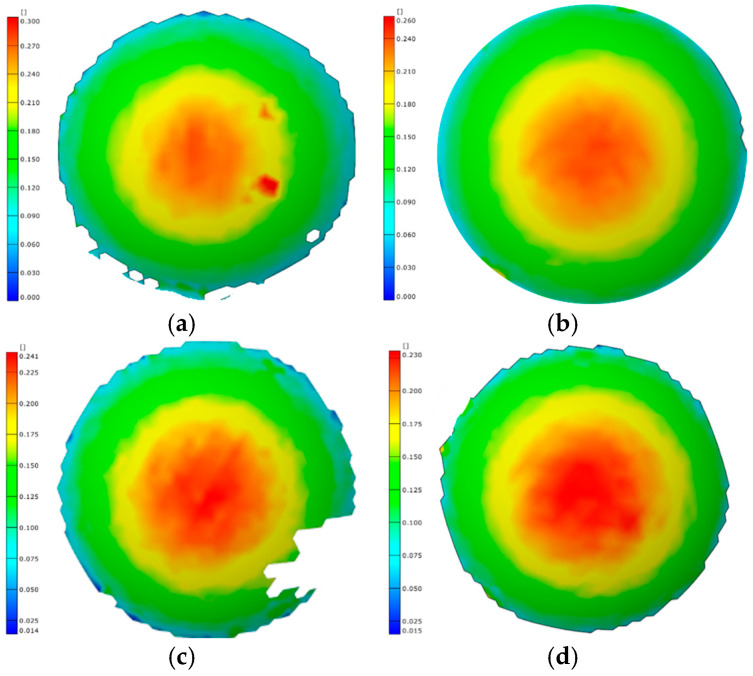
Strain distribution at bulging height of 20 mm at different temperatures: (**a**) 350 °C; (**b**) 375 °C; (**c**) 400 °C; and (**d**) 425 °C.

The strain distributions of bulged specimens formed by the rapid deflation process at different temperatures with a bulging height of 20 mm are shown in Figure 16. For all specimens, the strain exhibits a uniform annular distribution. The maximum strain is located at the apex region of the bulged bottom. The strain gradually decreases from the apex toward the perimeter region. Therefore, under different temperature conditions, the rapid deflation process produces bulged specimens with a highly uniform strain distribution.

**Figure 17:**
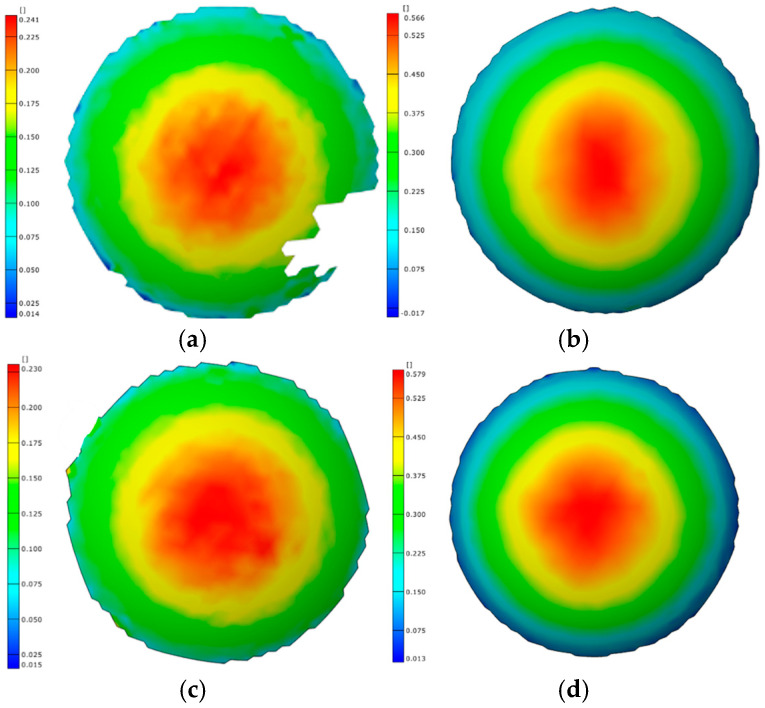
Strain distribution at different bulging heights: (**a**) 20 mm-400 °C; (**b**) 30 mm-400 °C; (**c**) 20 mm-425 °C; and (**d**) 30 mm-425 °C.

Under the same temperature conditions, bulged specimens formed to different bulging heights by the rapid deflation process are shown in Figure 17. The corresponding strain values are summarized in Table 5. In all cases, the maximum principal strain is located at the apex region of the bulged specimens. This indicates that the location of maximum deformation during the bulging process consistently occurs at the apex. The deformation magnitude gradually decreases along the radial direction. Regardless of whether the bulging stage is in the early or late phase, the strain distribution remains circumferentially uniform. As a result, the sheet undergoes uniform deformation, indicating favorable formability.

## 5. Conclusions

This study focuses on the hot-state rapid gas pressure forming of 2A12 aluminum alloy sheets, where an experimental setup for hot-state rapid gas pressure forming was established, and experiments were conducted using conventional rapid inflation with central and perimeter gas inlets as well as the hot-state rapid deflation process. The experimental results were compared, and the profiles and strain distributions of the high-speed bulged sheets were analyzed to investigate the wall thickness distribution patterns. The following conclusions were drawn:

1. At 400 °C, under different bulging pressures, compared to the rapid inflation process, the use of the rapid deflation process results in a higher mold-fit accuracy and smaller corner radii for the bulged components, indicating higher forming precision. Additionally, as the bulging pressure increases, the forming precision improves.
2. Under consistent bulging pressure, temperature, and sheet forming height, compared to the sheet formed using the rapid inflation process, the bulged components formed using the rapid deflation process exhibit a more uniform wall thickness distribution. Moreover, as the temperature increases, the wall thickness uniformity in the material bulging area improves. At higher forming heights, under the same conditions, the differences in wall thickness distribution become larger, and the thinning becomes more pronounced.
3. Under the same bulging pressure and temperature, the forming limit of aluminum alloy sheets formed using the rapid deflation process is higher than that of aluminum alloy sheets formed using either center rapid inflation or perimeter rapid inflation processes.
4. The strain analysis of the bulged components indicates that, when forming spherical-bottomed components, under different temperature and bulging height conditions, the maximum strain of the bulged components from both forming processes occurs at the apex region, where it is evenly distributed circumferentially and gradually decreases radially.

## Figures and Tables

**Table 1:** Chemical composition of 2A12 aluminum alloy sheet (wt%) [33].

Cu	Mg	Mn	Fe	Si	Zn	Ti	Ni	Al
4.78	1.56	0.57	0.24	0.11	0.2	0.1	0.1	Balance

**Table 2:** Experimental scheme of sheet hot gas bulging.

Pressure Applying Method	Deformation Temperature (°C)	Bulging Pressures (MPa)	Formed Insert Block	Bulging Height (mm)
Perimeter rapid inflation	400	6, 9, 12	Radius 35 mm flat insert piece	20
Center rapid inflation	400	6, 9, 12	Radius 35 mm flat insert piece	20
Rapid deflation	400	6, 9, 12	Radius 35 mm flat insert piece	20
Perimeter rapid inflation	350, 375, 400, 425, 450	10	Radius 35 mm spherical defect insert piece	20, 30
Center rapid inflation	350, 375, 400, 425, 450	10	Radius 35 mm spherical defect insert piece	20, 30
Rapid deflation	350, 375, 400, 425, 450	10	Radius 35 mm spherical defect insert piece	20, 30

**Table 3:** Comparison of the die-fitting degree between rapid deflation and perimeter rapid inflation under different bulging pressures.

Bulging Pressure (MPa)	Die-Fitting Degree (Rapid Deflation)	Die-Fitting Degree (Perimeter Rapid Inflation)
6	85.4%	83.4%
9	87.5%	85.5%
12	89.5%	87.5%

**Table 4:** Summary of strain experimental results for a bulging height of 30 mm under different bulging methods and deformation temperatures.

Temperature	Perimeter Rapid Inflation				Rapid Deflation			
	Cracking Occurrence	Whether the Target Bulging Height Was Achieved	Maximum Strain Value	Location of Maximum Strain	Cracking Occurrence	Whether the Target Bulging Height Was Achieved	Maximum Strain Value	Location of Maximum Strain
350 °C	Yes	No	0.55	Edge at the cracking location	Yes, but with lesser size.	No	0.48	Edge at the cracking location
375 °C	Yes	No	0.53	Edge at the cracking location	Yes, but with lesser size.	No	0.62	Edge at the cracking location
400 °C	Yes	No	0.51	Edge at the cracking location	No	Yes	0.56	Apex of the bulged specimen
425 °C	Yes	No	0.57	Edge at the cracking location	No	Yes	0.58	Apex of the bulged specimen
450 °C	Yes	No	0.44	Edge at the cracking location	No	Yes	0.44	Apex of the bulged specimen

**Table 5:** Maximum principal strain values of bulged specimens formed to different bulging heights using the rapid deflation process.

Temperature (°C)	Maximum Principal Strain at a Bulging Height of 20 mm	Maximum Principal Strain at a Bulging Height of 30 mm
400	0.24	0.56
425	0.23	0.58

## Data Availability

The original contributions presented in this study are included in the article. Further inquiries can be directed to the corresponding author.
